# A cross-sectional study on the first-in-human trials of anticancer drugs in Japan and the United States and the probability of approval

**DOI:** 10.1007/s10147-025-02849-4

**Published:** 2025-08-06

**Authors:** Akari Mukaida, Hideki Maeda

**Affiliations:** Regulatory Science, Faculty of Pharmacy, Meiji Pharmaceutical University, 2-522-1, Noshio, Kiyose-City, Tokyo, 204-5255 Japan

**Keywords:** First-in-human, Phase I, Approval, Oncology, Japan, United States

## Abstract

**Background:**

This study aimed to examine the characteristics of First-in-human (FIH) trials conducted in Japan and the US and whether the probability of approval for pharmaceuticals that had undergone FIH trials differs in the two countries.

**Methods:**

FIH trials of anticancer drugs initiated between 2007 and 2017 were investigated in this study. The trials were searched using ClinicalTrials.gov.

**Results:**

There were 22 FIH trials conducted in Japan and 261 in the US. Of these, six drugs (27.2%) were approved in Japan and 27 (10.3%) were approved in the US, indicating that the probability of approval was significantly higher for FIH trials conducted in Japan than in the US. Comparison of the characteristics of FIH trials between Japan and the US, showed that 81.8% of the FIH trials conducted in Japan were sponsored by the top 20 pharmaceutical companies, whereas 55.6% in the US were sponsored by non-top 20 companies (*P* = *0*.003, Chi-square test). The number of patients was higher in Japan than in the US (*P* = *0*.044). Further, all of the trials conducted in Japan were multiregional clinical trials in collaboration with other countries such as Europe and the US, whereas 49.0% of the trials in the US were conducted in the US alone (*P* < *0*.001).

**Conclusion:**

We inferred that the FIH trials conducted in Japan are multiregional clinical trials by major pharmaceutical companies with Europe and the US, and are conducted with drugs that are expected to have a high probability of successful approval.

**Supplementary Information:**

The online version contains supplementary material available at 10.1007/s10147-025-02849-4.

## Introduction

A first-in-human (FIH) trial is a mandatory step in the clinical development of a new pharmaceutical product aimed at obtaining manufacturing and sales approval [1, 2]. Further, it is crucial for the development of pharmaceutical products to advance promising drug candidate compounds from preclinical studies to FIH clinical trials [2]. At the stage of initiating FIH trials, several factors, such as their effects on humans, remain unknown; hence, FIH trials must be conducted carefully and with a limited number of individuals, and clinical trials must be conducted at medical institutions with advanced equipment and competent personnel [3]. FIH trials are usually conducted on healthy adults to investigate the safety of pharmaceutical products. However, for anticancer drugs, clinical trials are conducted on patients with cancer, and the purpose of proof of concept (PoC) is often taken into account [1, 4].

Several Japanese pharmaceutical companies used to conduct early-stage clinical trials such as FIH trials and PoC trials in Japan. However, from approximately the year 2000, drug lag has become a problem in Japan, and the utilization of overseas data and bridging strategies have become popular as drug development strategies, and clinical development in Japan has been following that in Europe and the US [5]. Furthermore, Japanese pharmaceutical companies are now targeting the US market rather than the Japanese market, and consider launching their products in the US to be a core part of their development strategy. Thus, the companies moved their development headquarters from Japan to the US, where the Food and Drug Administration (FDA) is located [6]. In terms of pharmaceutical regulations for development, with the progress of the International Council for Harmonisation of Technical Requirements for Pharmaceuticals for Human Use (ICH), several guidelines for multiregional clinical trials have been issued [7–9]. Pivotal trials such as Phase 3 trials are now being conducted as multiregional clinical trials, with Japan increasingly participating in such trials, and Japan now submits applications at the same time as Europe and the US. Furthermore, the review period has been shortened, and drug lag can be resolved [10]. However, although global clinical development has occurred simultaneously, FIH trials are still rarely conducted in Japan and are usually conducted in Europe and the US [4]. Since 2010, demand for Japan-originated FIH trials is growing, and momentum has been building in industry, government and academia for the necessity to establish an implementation system in Japan [11–13]. In Apr 2012, a guidance regarding FIH trials in Japan called “Guidance for ensuring safety in first-in-human studies in drug development” was issued [14], and the infrastructure for FIH trials at medical institutions has been developed. It is believed that FIH trials are gradually being conducted more frequently in Japan; however, there are still several unknowns regarding the actual situation of FIH trials in Japan.

Therefore, this study focused on Japan and the US as countries where FIH trials are conducted, and aimed to clarify the characteristics and background of FIH trials of anticancer drugs conducted in these two countries. Furthermore, for understanding differences in the characteristics of FIH trials in the two countries, we aimed to examine whether there is a difference in the probability of drug approval between cases where the FIH trial of the drug is conducted in Japan and in the US.

## Materials and methods

### Selection of drugs and database for research

This study covered a 10 years period from Jan 2007 to Dec 2017, focusing on a ± 5 years period around 2012, when the guidance on FIH trials was issued in Japan. FIH trials of anticancer drugs initiated in Japan or the US during that 10 years period were surveyed. ClinicalTrials.gov (https://clinicaltrials.gov/), a clinical trial registry database, was used to search for FIH trials. To extract FIH trial data of anticancer drugs, we first conducted a search on ClinicalTrials.gov using the following search formula:

- Condition/disease: “Oncology”.
- Other terms: “first in man” or “first-in-human”.
- Study Status: All studies.
- Study Phase: Phase 1.
- Study type: Interventional.
- Location: “Japan” or “United States”.

The above data extraction using ClinicalTrials.gov was performed on June 1, 2024.

### Data collection and abstraction

Information on the background of each drug, its regulatory background and the application for drug approval was collected from public sources. Information of the drugs approved in Japan was collected from the website of the Pharmaceuticals and Medical Devices Agency (PMDA) website (https://www.pmda.go.jp/PmdaSearch/iyakuSearch/). Information of the drugs approved in US was collected from Drugs@FDA (the website of the FDA) (http://www.accessdata.fda.gov/scripts/cder/drugsatfda/). From the public information, we extracted detailed information of clinical trials, as well as information on drug background, regulatory background and application for approval, and created our own database. This study was conducted in accordance with Strengthening the Reporting of Observational Studies in Epidemiology (STROBE) reporting guidelines [15] for cross-sectional studies.

### Statistical analysis

We used descriptive statistics to characterize the new drugs and their indications. We used the chi-square test for trend comparisons of regulatory characteristics between public knowledge-based application and other applications. All statistical tests were two-tailed, and *P *values of less than 0.05 were used to denote statistical significance. All analyses were conducted using analytical tools of Microsoft Excel 2019 MSO.

## Results

### FIH trials investigated

**Fig. 1:**
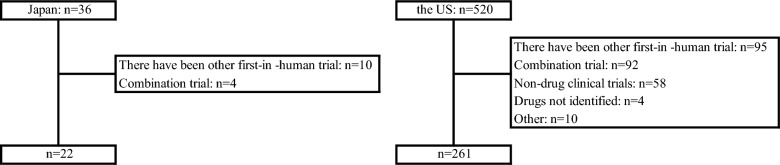
Flowchart of FIH trials investigated in the study

A search on ClinicalTrials.gov on June 1, 2024 found 36 FIH trials in Japan and 520 in the US over the 10 years study period from Jan 2007 to Dec 2017. Each trial was reviewed closely and 14 trials in Japan and 259 trials in the US were excluded due to reasons such as “there is another FIH trial”, “the pharmaceutical product cannot be identified”, “it is an FIH trial of a combination trial” and “it is not an FIH trial of a pharmaceutical product”. Ultimately, 22 FIH trials in Japan and 261 in the US were investigated in this study. The FIH trials investigated in this study are shown in Fig. 1.

**Fig. 2:**
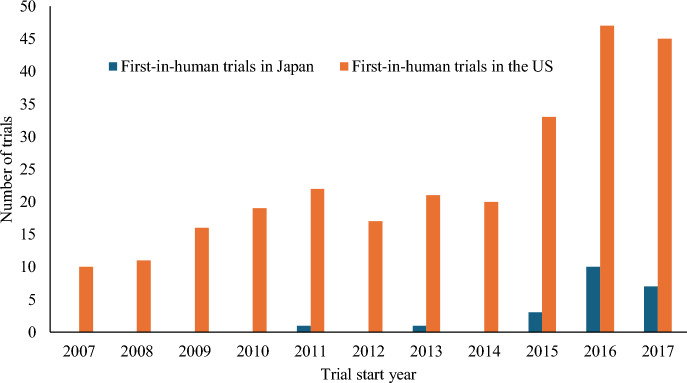
Number of oncology FIH trials from 2007 to 2017 in Japan and the US

We examined FIH trials from 2007 to 2017, with a primary focusing on those conducted in 2012, when the FIH guideline was issued, and the years immediately before and after. Additionally, the change over time in the number of FIH trials conducted in Japan or the US is shown in Fig. 2. FIH trials increased annually in Japan and the US; however, every year, the US conducted more number of FIH trials than Japan. Our investigation revealed that the number of FIH trials Japan and the US increased by 12 and 70 trials, respectively, in 2018 (data not shown). Moreover, the number of FIH trials in both countries continued to increase during this decade.

### Comparison of the characteristics and background of FIH trials between Japan and the US

Table 1 lists the clinical characteristics of the FIH trials investigated in this study. With regard to sponsorship, a high percentage of FIH trials conducted in Japan were sponsored by the top 20 pharmaceutical companies at 81.8%, whereas more than half of the FIH trials conducted in the US (55.6%) were sponsored by non-top 20 pharmaceutical companies. The difference in sponsors was significant between FIH trials conducted in Japan and the US (*P* = *0*.003, Chi-square test). In terms of the number of patients, while most FIH trials in Japan were trials with 100–300 subjects or large-scale trials with 500 or more subjects, most FIH trials in the US were conducted with 50 or fewer subjects (*P* = 0.044, Chi-square test).

**Table 1:** Characteristics of oncology FIH trials from 2007 to 2017 in Japan or the US

Items		Japan		the US		*P *value
		*N*	%	*N*	%	
Total number		22	100.0	261	100.0	–
Mechanism of action	Cytotoxic drug	0	0.0	3	1.1	0.860
	Molecularly-targeted drug	10	45.5	134	51.3	
	Antibody preparation	9	40.9	85	32.6	
	Antibody drug conjugate	3	13.6	25	9.6	
	Immunotherapy	0	0.0	7	2.7	
	Hormonal drug	0	0.0	1	0.4	
	Others	0	0.0	6	2.3	
Cancer type	Solid cancer	16	72.7	210	80.5	0.063
	Hematologic cancer	3	13.6	28	10.7	
	Solid cancer and Hematologic cancer	3	13.6	23	8.8	
Sponsor size/type	Top 20 company	18	81.8	116	44.4	0.003
	Other company	4	18.2	132	50.6	
	Investigator/government	0	0.0	13	5.0	
Sponsor capitol	Domestic company	2	9.1	13	5.2	0.450
	Foreign company	20	90.9	235	94.8	
Number of patient	50	3	13.6	106	40.6	0.044
	51–100	2	9.1	59	22.6	
	101–200	7	31.8	47	18.0	
	201–300	4	18.2	24	9.2	
	301–400	0	0.0	7	2.7	
	401–500	1	4.5	4	1.5	
	501	5	22.7	14	5.4	

We also investigated the countries where FIH trials of anticancer drugs in Japan or the US were conducted. More than 80% of the FIH trials in Japan were also conducted in the US and Europe as multiregional clinical trials. In contrast, about half of the FIH trials in the US (49.0%) were conducted in the US alone, and the percentage of trials conducted as multiregional clinical trials was significantly lower than in Japan (*P* < *0*.001, Chi-square test) (Table 2).

**Table 2:** Location of oncology FIH trials from 2007 to 2017 in Japan or the US

Japan			The US			*P *value
Location	*N*	%	Location	*N*	%	
Japan	22	100.0	the US	261	100.0	< 0.001
Japan + the US	19	86.4	the US + Japan	20	7.7	
Japan + Europe^*1^	18	81.8	the US + Europe^*1^	103	39.5	
Japan + China	4	18.2	the US + China	7	2.7	
Japan + others^*2^	18	81.8	the US + others^*3^	107	41.0	
Japan only	0	0.0	the US only	128	49.0	

^*^1: United Kingdom, France, Italy, Spain, Germany, Switzerland

^*^2: others: Canada;12, Australia;11, Singapore;9, Korea;8, Chinese Taipei;8

^*^3: others: Canada;46, Australia;37, Netherlands;29, Korea;28, Belgium;26, Singapore;13, Denmark;12

### Comparison of probability of approval between Japan and the US

We examined whether the drugs investigated in the FIH trials conducted in Japan or the US were approved in Japan or the US. The results are shown in Table 3. Of the 22 FIH trials conducted in Japan, six drugs (27.2%) were approved in Japan or the US, whereas of the 261 trials conducted in the US, 27 drugs (10.3%) were approved in Japan or the US, indicating that the probability of approval was significantly higher for FIH trials conducted in Japan than those in the US (*P* = 0.048, Chi-square test).

**Table 3:** Status of approval by FDA or PMDA on oncology drugs started first-in human trial from 2007 to 2017

Status	Japan		the US		*P *value
	*N*	%	*N*	%	
Approved	6	27.2	27	10.3	0.048
Withdrawal	1	4.5	7	2.7	
Under development/unknown	15	68.2	227	87.0	
Total	22	100.0	261	100.0	–

For reference, Supplemental Table 1 lists the clinical characteristics of drugs approved after FIH trials in Japan or the US. No significant differences were found between Japan and the US in the items examined.

## Discussion

Considering the history of FIH trials in Japan, few FIH trials were conducted in Japan around 2010. At that time, Japan had the capability to create pharmaceutical products through basic research; however, it was pointed out that Japan was unable to conduct clinical research trials and lacked the capacity to conduct FIH trials. This situation was said to show that a “valley of death” existed between basic and clinical research [6, 16]. It was thought that industry, government, and academia each had their own issues to address in conducting FIH trials [6]. The challenges were as follows: in academia, the infrastructure for conducting FIH trials was not in place and competent personnel to conduct FIH trials were not available. There was also an opinion that in order to conduct FIH trials in Japan, academia should clearly state areas in which Japan excels and areas in which it leads the world, and conduct FIH trials in those areas. With regard to regulatory authorities, when before conducting FIH trials in Japan, more data from non-clinical studies, such as toxicity studies, are required in Japan than overseas, and that the requirements by the regulatory authorities for conducting FIH trials were unclear. There have been criticisms that even Japanese pharmaceutical companies place too much emphasis on Europe and the US, and that early-stage clinical trials in development are led by Europe and the US at foreign mega-pharmaceutical companies, making it impossible for Japanese branch offices to explain the rationale for conducting FIH trials in Japan to their European and US headquarters. Since then, many parts of these issues, challenges and comments have been addressed by industry, government and academia; however, solutions have not been attained. In academia, systems and facilities for FIH trials have been established, particularly at cancer centers, and infrastructure has been rapidly developed to a level similar to that in Europe and the US. In addition, diseases and research areas such as gastric cancer, liver cancer, lung cancer and mesothelioma, which are common diseases in Japan, have been disseminated to the world as the areas of expertise of Japan [6]. From the regulatory perspective, in addition to the FDA’s guidance on FIH trials and the guidance of the European Medicines Agency on high-risk drugs for FIH trials, Japan also issued the FGuidance for ensuring safety in first-in-human studies in drug development” in Apr 2012 [14], bringing the three regulatory bodies together. The non-clinical studies required for FIH trials were also standardized to international standards by ICH-S9 [17]. The above changes in the situation have caused pharmaceutical companies to rethink the implementation of FIH trials and early exploratory clinical trials in Japan. Especially in fields such as oncology, it is now commonplace for global FIH trials to be conducted under the three regulatory bodies. In addition, the recent depreciation of the yen has also had an impact, giving the impression that the implementation of FIH trials in Japan is on the rise. In our study, two drugs (9.1%) that underwent FIH in Japan received Fast Track designation from the FDA. Additionally, four drugs (18.2%) received Breakthrough Therapy designation from the FDA. This suggests that drugs that undergo FIH in Japan also contribute to global development. Furthermore, previous reports have shown overlaps between special pharmaceutical measures in Japan and the US. Therefore, the implementation of FIH in Japan may impact the US [18].

To the best of our knowledge, our study is the first to comprehensively review FIH trials in Japan, and compare them with those in the US. In the present study, a comparison of the number of FIH trials between the two countries showed that the number of FIH trials conducted in the US was 10 times higher than those in Japan. The change in the number of trials over time showed an increasing trend in both Japan and the US. However, every year, the US conducted more FIH trials than Japan. Comparing the characteristics of FIH trials in Japan and the US, many trials in Japan were conducted by the top 20 sales pharmaceutical companies, whereas many trials in the US were conducted by non-top 20 pharmaceutical companies. We inferred that major pharmaceutical companies conduct FIH trials in Japan, whereas they are conducted in the US by bio-ventures and other companies. The number of patients was significantly higher in FIH trials conducted in Japan than those in the US. Comparison of the regions where the FIH trials were conducted revealed that, most of the FIH trials conducted in Japan were also conducted in the US and Europe, whereas the percentage of FIH trials conducted in the US that were being conducted in other countries was significantly lower. We inferred that Japan is currently participating in FIH trials that are conducted as multiregional clinical trials. The present study also examined the probability of drug approval following FIH trials conducted in Japan and the US. Previous studies in Europe and the US have shown that the probability of approval for pharmaceutical products that have undergone FIH trials is as low as 8–10% [19, 20]. Another study in the US examined the likelihood of approval for anticancer drugs that had undergone Phase I trials between 2011 and 2020, and found it to be 5.3% [21]. Studies outside Europe and the US showed that out of the FIH trials conducted in China between 2012 and 2020, 5.8% investigated drugs that were ultimately approved by the National Medical Products Administration of China [22]. Thus, it can be seen that the probability of approval following FIH trials is approximately 5–10%. In the present study, the probability of approval for drugs in the FIH trials conducted in the US was 10.3%, similar to that of previous studies. However, the FIH trials conducted in Japan showed a high probability of obtaining drug approval (27.2%). Considering the results of our study, we speculated that the reason for this is that most of the FIH trials conducted in Japan are FIH trials by major pharmaceutical companies, such as top 20 sales companies, and many are conducted as multiregional clinical trials with drugs that are expected to have a certain high probability of successful approval. Further, it was suggested that FIH trials conducted in the US are often conducted by small-scale companies such as bio-ventures in the US alone, with a small number of patients. Initially, we hypothesized that the FIH trials conducted in Japan would have a low probability of approval since the infrastructure had only been established recently and it had a short history; however, we found that this was not the case. We believe that, Japan can contribute to improving the global approval probability of anticancer drugs through international cooperation, e.g., participating in multicentre clinical trials.

The present study has several limitations. First, we used ClinicalTrial.gov as our search database for clinical trials; however, trials and pharmaceutical products not registered with it were not investigated. Second, we investigated approval status based on information from public sources such as company websites, but pharmaceutical companies may not have announced the discontinuation of new drug development, and these drugs may have been classified as under development. Third, the probability of approval may be influenced by several potential confounders outside the scope of this study (e.g., cancer type, mechanism of action, investigational site selection, and size of company etc.). Finally,, since only anticancer drugs are included in this study, FIH trials in other areas are expected to yield different results.

## Conclusion

We investigated FIH trials of anticancer drugs initiated in Japan or the US between 2007 and 2017. The number of FIH trials in Japan and the US increased annually; however, we found that the backgrounds including “sponsors”, “number of patients” and “regions where FIH trials are conducted” are different between Japan and the US. In addition, the probability of approval was higher for anticancer drugs that underwent FIH trials in Japan than for those in the US. FIH trials in Japan and the US exhibited differences in characteristics. Further, the probability of approval was higher for anticancer drugs that had undergone FIH trials in Japan than those in the US. Based on these, it was inferred that the FIH trials conducted in Japan are clinical trials by major pharmaceutical companies, are conducted as multiregional clinical trials in collaboration with Europe and the US, and with drugs that are expected to have a certain high probability of successful approval.

## Supplementary Information

Below is the link to the electronic supplementary material.

Supplementary file1 (XLSX 11 KB)

## Data Availability

Data available on request from the researchers after approval by the corresponding author.
